# Immobilized Soybean Peroxidase Hybrid Biocatalysts for Efficient Degradation of Various Emerging Pollutants

**DOI:** 10.3390/biom11060904

**Published:** 2021-06-17

**Authors:** Rana Morsi, Khadega A. Al-Maqdi, Muhammad Bilal, Hafiz M. N. Iqbal, Abbas Khaleel, Iltaf Shah, Syed Salman Ashraf

**Affiliations:** 1 Department of Chemistry, College of Science, United Arab Emirates University, Al Ain P.O. Box 15551, United Arab Emirates; 201350167@uaeu.ac.ae (R.M.); 200935138@uaeu.ac.ae (K.A.A.-M.); abbask@uaeu.ac.ae (A.K.); altafshah@uaeu.ac.ae (I.S.); 2 Huaiyin Institute of Technology, School of Life Science and Food Engineering, Huaian 223003, China; bilaluaf@hyit.edu.cn; 3 Tecnologico de Monterrey, School of Engineering and Sciences, Monterrey 64849, Mexico; hafiz.iqbal@tec.mx; 4 Department of Chemistry, College of Arts and Sciences, Khalifa University, Abu Dhabi P.O. Box 127788, United Arab Emirates

**Keywords:** biocatalysts, soybean peroxidase, emerging pollutants, photocatalysts, immobilization, redox mediator, metal-oxide catalysts

## Abstract

In the present study, soybean peroxidase (SBP) was covalently immobilized onto two functionalized photocatalytic supports (TiO_2_ and ZnO) to create novel hybrid biocatalysts (TiO_2_-SBP and ZnO-SBP). Immobilization caused a slight shift in the pH optima of SBP activity (pH 5.0 to 4.0), whereas the free and TiO_2_-immobilized SBP showed similar thermal stability profiles. The newly developed hybrid biocatalysts were used for the degradation of 21 emerging pollutants in the presence and absence of 1-hydroxy benzotriazole (HOBT) as a redox mediator. Notably, all the tested pollutants were not equally degraded by the SBP treatment and some of the tested pollutants were either partially degraded or appeared to be recalcitrant to enzymatic degradation. The presence of HOBT enhanced the degradation of the pollutants, while it also inhibited the degradation of some contaminants. Interestingly, TiO_2_ and ZnO-immobilized SBP displayed better degradation efficiency of a few emerging pollutants than the free enzyme. Furthermore, a combined enzyme-chemical oxidation remediation strategy was employed to degrade two recalcitrant pollutants, which suggest a novel application of these novel hybrid peroxidase-photocatalysts. Lastly, the reusability profile indicated that the TiO_2_-SBP hybrid biocatalyst retained up to 95% degradation efficiency of a model pollutant (2-mercaptobenzothiazole) after four consecutive degradation cycles.

## 1. Introduction

Emerging pollutants (Eps) are a new class of organic chemicals that are increasingly detected in water bodies. These pollutants are referred to as synthetic chemicals or naturally occurring compounds that are found in the natural environment without being monitored or regulated and have a significant environmental impact [1,2]. Emerging pollutants comprehend a wide range of various compounds and their transformation products such as nonsteroidal anti-inflammatory drugs, analgesics, antibiotics, textile dyes, hormones, personal care products, and pesticides, among other potentially toxic substances [3]. Though the concentration of Eps in the environmental matrices ranges from ng/L to few hundreds of μg/L [4,5], these are recognized to cause serious ecological and physiological threats such as interfering with the endocrine system, reproductive impairments, physical abnormalities, congenital disorders, and feminization of some fish species [6]. Due to these undesirable and deleterious effects on human health and the ecosystem, research on emerging pollutants has prioritized the focus of researchers and environmental engineers around the globe. 

Though various chemical and physical methods have been proposed for the treatment of contaminated wastewater [7,8,9,10,11], several limitations such as high cost, generation of sludge, and toxic by-products hinder their application [12,13,14,15,16]. Hence, the development of new, efficient, greener, and environmentally responsible technologies for wastewater remediation remain a principal endeavor for researchers, industrial chemists, and the scientific community. A biological approach using oxidoreductase enzymes is capable of degrading a vast array of structurally diverse pollutants and thus appears a promising area of research in water treatment [12]. Numerous enzyme systems have been employed over the last few years for the efficient degradation of diverse organic pollutants leading to oxidize recalcitrant pollutants into smaller intermediates. The deployment of enzyme-based biocatalytic processes offers many advantages such as low energy input, non-toxicity, ability to operate under mild aqueous conditions, reduced amount of sludge generation, and can be applied over a wide range of pollutants [17]. 

Oxidoreductases (oxidases, peroxidases, dehydrogenases, and oxygenases) are the most widely investigated class of enzymes for the bioremediation of wastewater. These enzymes catalyze the oxidation–reduction-assisted biodegradation of various classes of hazardous organic pollutants including cresols, phenols, chlorinated phenols, herbicides, pesticides, dioxins, synthetic textile dyes, pharmaceuticals, and personal care products [18]. It is well-known that different oxidoreductases, such as peroxidases and laccases, can efficiently degrade a number of organic pollutants [17,19,20,21]. For example, recently, Mukherjee et al. reported more than 95% degradation of 4,4′-methylenedianiline (MDA) by the SBP enzyme [22]. In another study, Rathner et al. showed the ability of horseradish peroxidase (HRP) to degrade more than 90% of 17α-ethinylestradiol (EE2) [23]. Many other studies described that different peroxidases such as lignin peroxidase (LiP) and manganese peroxidase (MnP) could also efficiently eliminate Eps from wastewater supplies. However, recent studies from our lab have shown that there are a number of emerging pollutants that are not degraded by peroxidases [19]. Nevertheless, an increasing body of literature seems to show that peroxidases hold promise for the remediation of organic pollutants.

Despite many advantages of enzymatic remediation, the high cost of catalytic enzymes, inability to reuse, possible conformational alterations, and enzyme activity loss under harsh environmental conditions (such as high temperatures, high/low pH values, and high ionic strength) are some of the challenges [12,24]. Many of these issues can be addressed by immobilizing enzyme onto different solid supports. Immobilization process converts the enzyme from its homogenous form to a heterogeneous catalyst (immobilized enzyme) to develop an immobilized biocatalyst [25]. The immobilized enzyme can be effectively used for the continuous degradation of high volumes of effluent [26,27]. Moreover, immobilization increases the long-term stability of enzymes as they become more resistant to degradation and denaturation and stable against harsh temperatures, pH, and pressure conditions [28,29]. Owing to advantages offered by enzyme immobilization compared to free enzymes, it can be deemed as a useful tool to markedly improving the catalytic properties of enzymes for large-scale applications.

The current study was carried out to expand the potential application of peroxidases by immobilizing SBP onto two photocatalytic supports (TiO_2_, ZnO) to address the limitations associated with scalability and non-reusability of these enzyme-based approaches.

The first objective of this study is to characterize the newly developed hybrid biocatalysts (TiO_2_-SBP and ZnO-SBP) in terms of surface morphologies, crystallinity, and pH and thermal stabilities. The second objective is to use the immobilized biocatalysts for the degradation of a mixture of twenty-one emerging pollutants in the presence and absence of a redox mediator. The third objective is to do a comparative degradation analysis between the free and immobilized peroxidases. The last objective of the current work is to investigate the recycling potential of immobilized SBP for emerging pollutant degradation. 

## 2. Materials and Methods

### 2.1. Reagents, Solvents, and Enzyme

Chemical standards of all emerging pollutants, glutaraldehyde, (3-aminopropyl) triethoxysilane (APTES), and photocatalysts (TiO_2_ and ZnO) were purchased from Sigma-Aldrich (Fremont, CA, USA). Solvents used in LC-MS such as water (LC-MS grade), formic acid, acetonitrile, and hydrogen peroxide (30% *w*/*v*) were also supplied by Sigma-Aldrich (St. Louis, MO, USA). Cellulose acetate syringe filters were procured locally from Medicom Distribution FZE (UAE). Bio-Research Products (North Liberty, IA, USA) furnished the SBP enzyme with a specific activity of 2700 IU/mg (1 mg/mL, 26 μM). All experiments were carried out using universal buffers consisting of 0.2 M K_2_HPO_4_ and 0.1 M citrate acid.

### 2.2. Immobilization of SBP onto Photocatalytic Supports

**Scheme 1:**
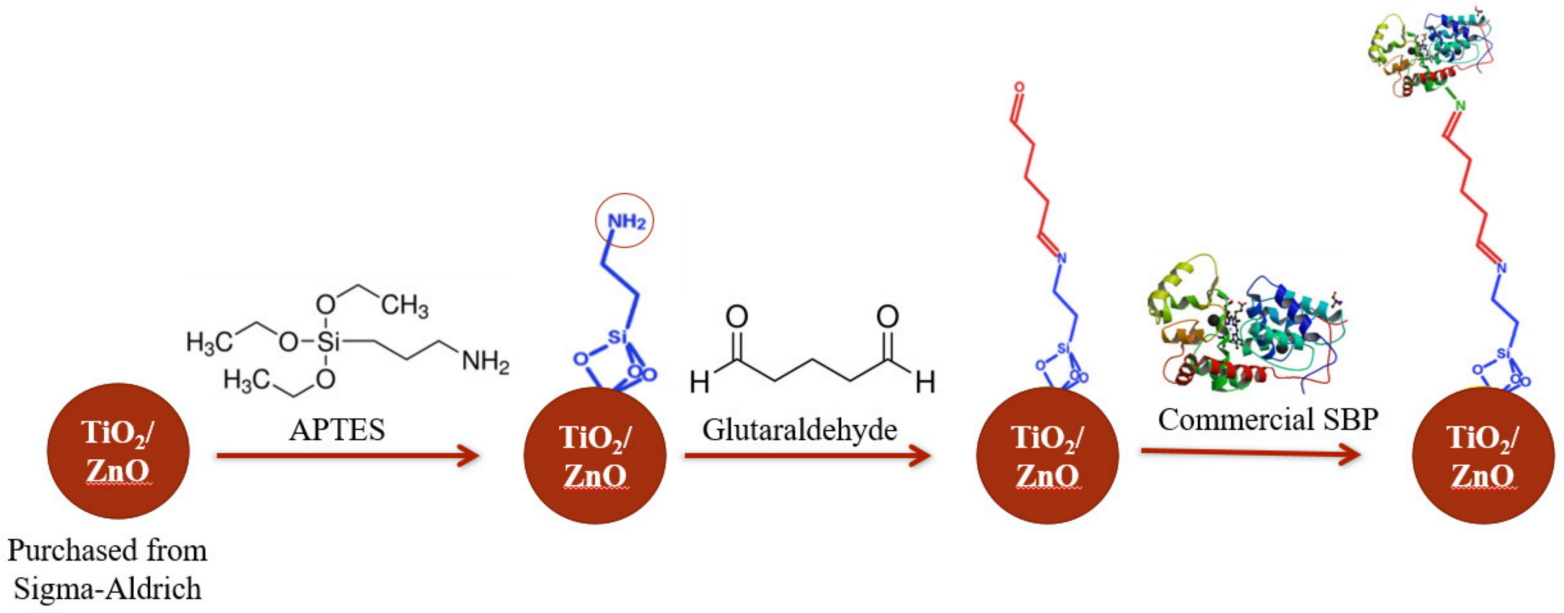
Summary of the preparation of the immobilized photo-catalysts (TiO_2_-SBP and ZnO-SBP).

Soybean peroxidase (SBP) enzyme was immobilized on the APTES-functionalized photocatalytic supports (TiO_2_ and ZnO particles), as depicted in Scheme 1 below, following a previously reported procedure [30]. Briefly, 3.0 g of commercially purchased TiO_2_ and ZnO were mixed with ethanol/water (1:1) solution, and the solution was left under nitrogen and sonicated for a few minutes. Then, 11.12 mL of APTES was added to the solution and stirred for 2 h at 40 °C. One gram of the produced TiO_2_-APTES and ZnO-APTES was added to a 100 mL solution of glutaraldehyde in phosphate buffer (0.1 M at pH = 7). The resultant mixtures were stirred in the dark for 1 h, and the products were filtered from the solution using Buchner funnel with vacuum suction. The separated products were incubated with 40 mL 5 µM SBP (which was in large molar excess with respect to aldehyde groups on functionalized TiO_2_-APTES) in phosphate buffer and allowed to stir overnight. The mixture was then filtered using Buchner funnel with vacuum suction, and the precipitated solid, which was obtained on the filter paper, was washed three times with phosphate buffer. After washing all solid particles were collected and preserved at 4 °C until further analysis. A side-by-side comparison (based on enzyme activity) indicated that about twice as much SBP was immobilized on TiO_2_ (per mg) as compared to ZnO.

### 2.3. Characterization of Photocatalytic Supports and Immobilized Enzyme

The photocatalytic supports and the immobilized enzymes were characterized using scanning electron microscopy (SEM) and powder X-ray diffraction. The morphology of the photocatalysts (TiO_2_ and ZnO), functionalized photocatalysts (TiO_2_-APTES and ZnO-APTES), and the newly developed hybrid biocatalysts (TiO_2_-SBP, ZnO-SBP) were investigated using an FEI SEM Quanta Inspect S50 SEM. Images were recorded at a voltage of 25 kV and magnification of ×10,000 (Supplementary Figure S1). The crystalline structure of all the samples (photocatalysts, functionalized photocatalysts, and hybrid biocatalysts) was examined using a Shimadzu-6100 X-ray powder diffractometer with Cu-Kα radiation, with the data collected from 10°–70° at a rate of 2°/min. (Supplementary Figure S2). Appropriate functionalization of the metal-oxides was confirmed using FTIR (Supplementary Figure S3).

### 2.4. Effect of pH and Temperature on the Stability of Free and Immobilized SBP

The free and immobilized biocatalyst samples were incubated in varying buffer solutions (ranging from pH 2.0 to 8.0 citrate-phosphate universal buffer) and assayed for activity to examine the influence of pH on their catalytic activities. Likewise, the thermal stabilities of free and immobilized SBP at varying temperatures were evaluated by heating enzyme suspension in a water bath for 10 min at the specified temperature (ranging from 30 to 90 °C) at the optimum pH (Supplementary Figure S4). The heated samples (10 µL) were immediately added to 190 µL “master mix” containing buffer, ABTS, and H_2_O_2_, at room temperature and the activity of the biocatalysts was assayed using a microplate reader at room temperature (~25 °C) as described previously [31]. The residual activity (relative activity) was calculated as a percentage of the initial activity.

### 2.5. Degradation Potential of Emerging Pollutants by Free and Immobilized Enzymes 

A mixture of 21 emerging pollutants was prepared and treated with the free SBP and immobilized hybrid enzyme (TiO_2_-SBP, ZnO-SBP) with and without the addition of HOBT redox mediator to investigate the remediation potential of the enzyme/hybrid catalyst. The degradation experiments for free enzymes were carried as follows: 0.39 μM (40.5 U/mL) of SBP enzyme was added to a 2 ppm (or µg/mL) mixture of 21 emerging pollutants + 0.3 mM H_2_O_2_ + pH 4.0 universal buffer (We have previously shown that SBP needs more than 0.05 mM H_2_O_2_ for the efficient degradation of aromatic pollutants and is tolerant to up to 18 mM H_2_O_2_ [32,33]. For the experiments in which redox mediator was used, 0.1 mM of 1-hydroxy benzotriazole (HOBT) was also added to the mixture. The degradation experiments for immobilized enzymes were carried out in the exact same way as the free enzymes, but instead of using a liquid enzyme, 20 mg of each hybrid biocatalyst (TiO_2_-SBP or ZnO-SBP) was added to the mixture and 0.6 mM of H_2_O_2_ was used in place of 0.3 mM H_2_O_2_ (The 0.6 mM H_2_O_2_ for the immobilized hybrid was the minimum amount of H_2_O_2_ that was needed for efficient degradation of the pollutants—data not shown). The mixture components were allowed to react at room temperature for 30 min, filtered and analyzed using liquid chromatography with tandem mass spectrometry (LC-MSMS).

For the UV-photolytic degradation experiments, 5 mg of TiO_2_, ZnO, TiO_2_-SBP, or ZnO-SBP was added to 5 mL of 2 ppm mixture of 21 emerging pollutants + 0.3 mM H_2_O_2_ (when added) + pH 4.0 universal buffer. The samples were irradiated from a 1.5 cm distance using a UV lamp (UVGL-58, J-129, Upland, NJ, USA). The UV power output for the UV lamp was 6 W and 254 nm output mode was selectively used for these studies. Under these conditions, there was no warming up of the irradiated samples.

### 2.6. Degradation Analysis by LC-MSMS Method

After the treatment of 21 emerging pollutants by the free and immobilized enzyme (TiO_2_-SBP, ZnO-SBP), the samples were analyzed by the LCMSMS, using the multiple reaction monitoring (MRM) mode, as previously described in detail [19]. Before injection to the LCMS, all the samples were filtered using cellulose syringe filters with a 13 mm inner diameter and a pore size of 0.45 μm. A ZORBAX Eclipse plus C column (1.8 μm particle size, 2.1 mm inner diameter and 50 mm length) was used for the analysis. The column’s temperature was maintained at 35 °C, and eluted with a flow rate of 0.4 mL/min. The mass spectrometer detector used was a 6420 Triple Quadrupole mass detector (Agilent Technologies). Two mobile phases were used: mobile phase (A) aqueous solution of 0.1% formic acid and mobile phase (B) 100% acetonitrile. A gradient elution was used for chromatographic separation of analytes and started at: 100% A and 0% B for 2.5 min, followed by 0–80% gradient of B from 2.5 to 15 min, this was followed by 10% A and 90% B for 3 min, and finally 95% A and 5% B was eluted through the column for 2 min to equilibrate the column. Both positive and negative polarity modes were used in the electrospray ionization source in the LCMS system depending on the pollutant analyzed. In the LC-MSMS interface system, the drying N_2_ gas flow was 8 L/min, and its temperature was kept at 3000 °C. The nebulization N_2_ gas pressure was set at 45 psi, and the capillary voltage was maintained at 4000 V. The nitrogen gas was used for fragmentation in the dissociation cell and precursor along with the product was detected in multiple reaction monitoring (MRM) mode with different collision energies depending on the emerging pollutants being analyzed.

### 2.7. Recycling Ability of the Immobilized Biocatalyst

In order to evaluate the reusability of the immobilized enzyme, TiO_2_-SBP hybrid biocatalyst was repeatedly used to degrade 2-mercaptobenzothiazole (MBT) pollutant in four consecutive catalytic cycles. After each reaction cycle, TiO_2_-SBP was separated from the reaction mixture and reused for the next reaction. The degradation experiment comprised 20 mg TiO_2_-SBP, 2 ppm MBT, 0.6 mM H_2_O_2_, universal buffer (pH 4.0), and 0.1 mM HOBT as a redox mediator. The reaction was kept at room temperature for 30 min, 0.5 mL of the mixture was filtered and analyzed using LC-MSMS. The residual sample was centrifuged (5 min), the supernatant was discarded, and the pellet containing the immobilized enzyme was allowed to react for another cycle for 30 min.

### 2.8. Statistical Analysis

Each sample was prepared in triplicates, and each replicate was analyzed twice in the LC-MSMS in order to check the variability of the results obtained. All the data were analyzed using Student’s unpaired *t*-tests. Data were reported as group mean ± standard deviation, and significance for all statistical comparisons was set at *p* < 0.05.

## 3. Results and Discussion

### 3.1. Soybean Peroxidase-Catalyzed Degradation of 21 Eps

As mentioned earlier, many groups have previously shown that peroxidases and laccases can degrade a number of different emerging pollutants [17,19,20,21,22]. The current study was carried out to expand the potential application of peroxidases by immobilizing SBP onto photocatalyst supports to address the limitations associated with the scalability and non-reusability of these enzyme-based approaches.

**Figure 1:**
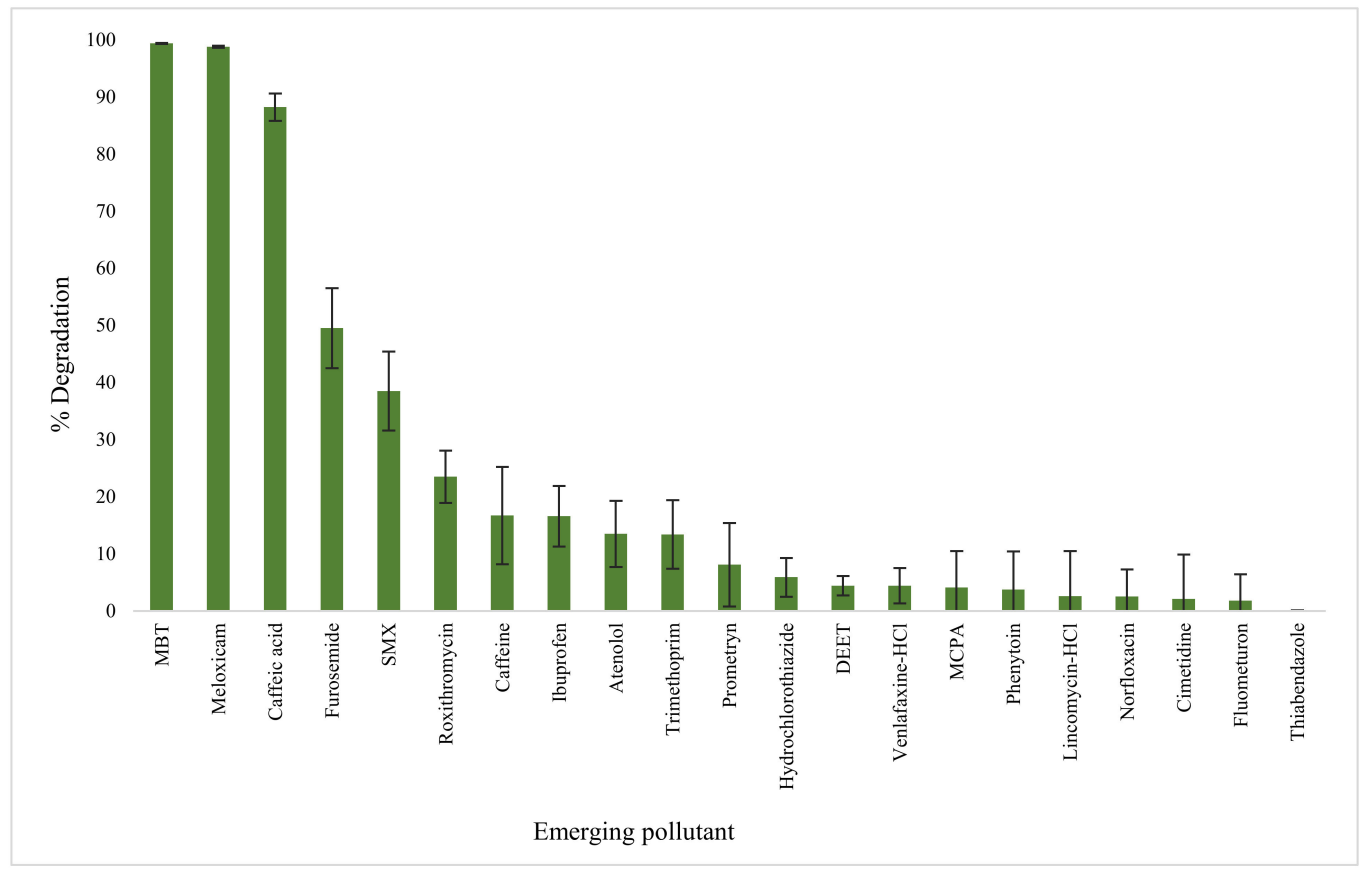
Degradation of 21 emerging pollutants by SBP + H_2_O_2_. [Pollutants] = 2 ppm, [Enzyme] = 0.39 μM, [H_2_O_2_] = 0.3 mM (0.1 mM added 3 times of 10 min interval), pH = 4.

**Figure 2:**
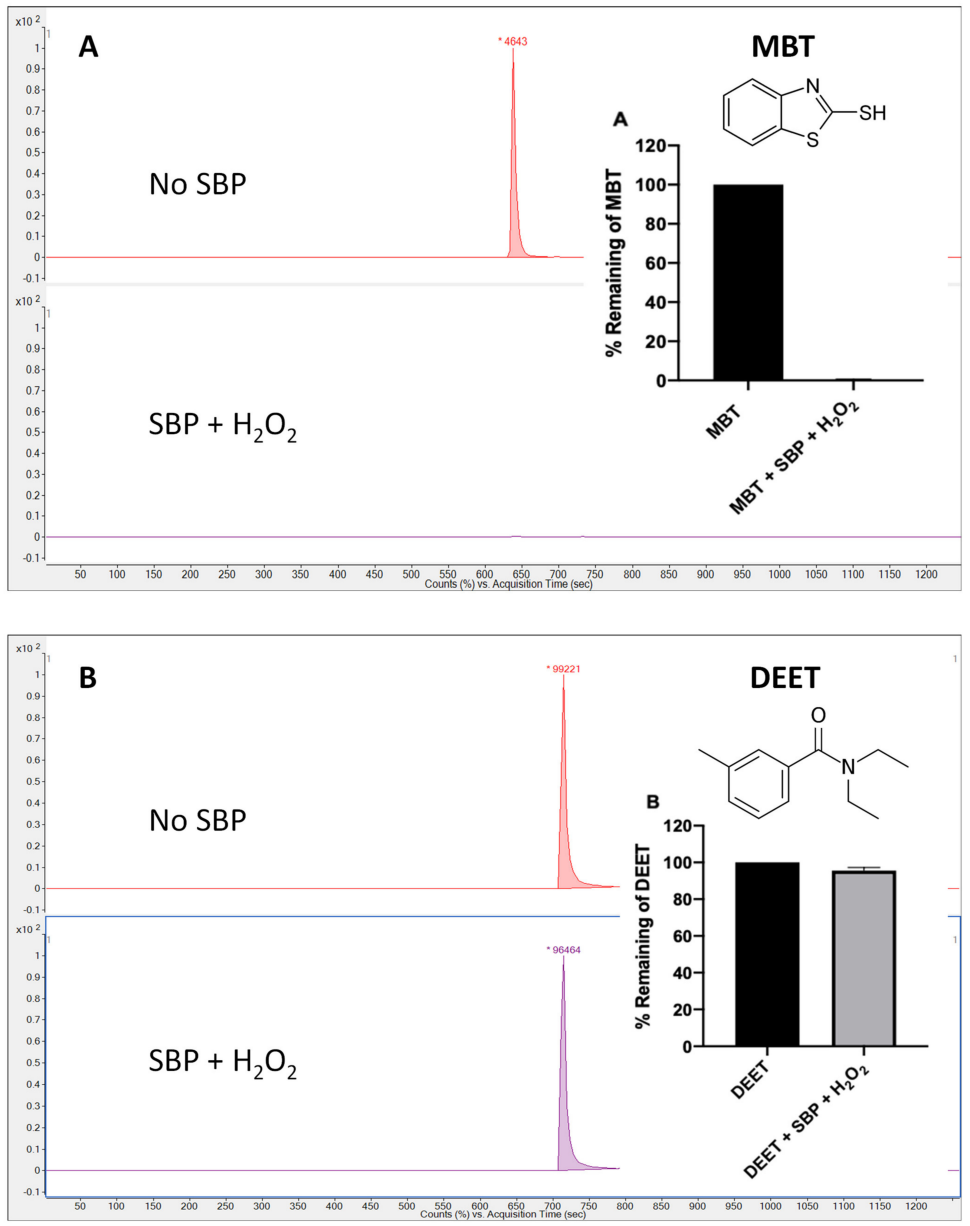
MRM scans of (**A**) MBT; and (**B**) DEET treated with SBP. [Pollutants] = 2 ppm, [Enzyme] = 0.39 μM, [H_2_O_2_] = 0.3 mM (0.1 mM added 3 times of 10 min interval), pH = 4.

A mixture of the 21 Eps was prepared and treated with an SBP enzyme to evaluate the ability of the peroxidase to degrade a diverse range of organic pollutants (Supplementary Table S1). As reported previously [19], we found that some of the Eps were not degraded by the enzyme treatment, as they were either only partially degraded or were resistant to degradation (less than 10% degradation). Figure 1 shows the results obtained when a mixture of these 21 Eps was treated with SBP (in the presence of H_2_O_2_) for 30 min. The percentage of degradation for each pollutant was calculated as previously described by using the MRM-based LC-MSMS method [19,34]. Based on the results obtained, it can be seen that the SBP was capable of efficiently degrading Mercaptobenzothiazole (MBT) (99.4% +/− 0.1), meloxicam (98.8% +/− 0.2), and caffeic acid (88.2% +/− 2.4). It could also catalyze the partial degradation of furosemide (49.5% +/− 7) and sulfamethoxazole (SMX) (38.5% +/− 6.9). A number of other pollutants were also degraded but to a much lower extent, for example, roxithromycin (23.5%), caffeine (16.7%), ibuprofen (16.6%), atenolol (13.5%), and trimethoprim (13.4%). The remaining 11 emerging pollutants showed insignificant degradation (less than 10%) by the SBP-mediated treatment. This dramatic difference between the SBP-mediated degradation of some Eps, such as MBT, and the inability of SBP to degrade others, such as DEET, is more clearly shown in Figure 2. Although not the focus of the current study, the inability of peroxidases such as SBP to degrade a number of emerging pollutants is an interesting observation, one that should be further examined by researchers in the field. Unfortunately, the field is full of selective examples of organic pollutants being enzymatically degraded. However, “negative results” (showing no degradation of some pollutants by peroxidases) are generally not reported. Although, the current approach of using a complex mixture of 21 different Eps is meant to simulate a real-life wastewater samples which would normally contain many different pollutants, the results obtained should be used with caution. For example, it is possible that the “lack of significant degradation” of a given EP may be due to potential inhibition or competition between other Eps and may not accurately reflect the inability of SBP to degrade them.

### 3.2. Requirement of a Redox Mediator

**Figure 3:**
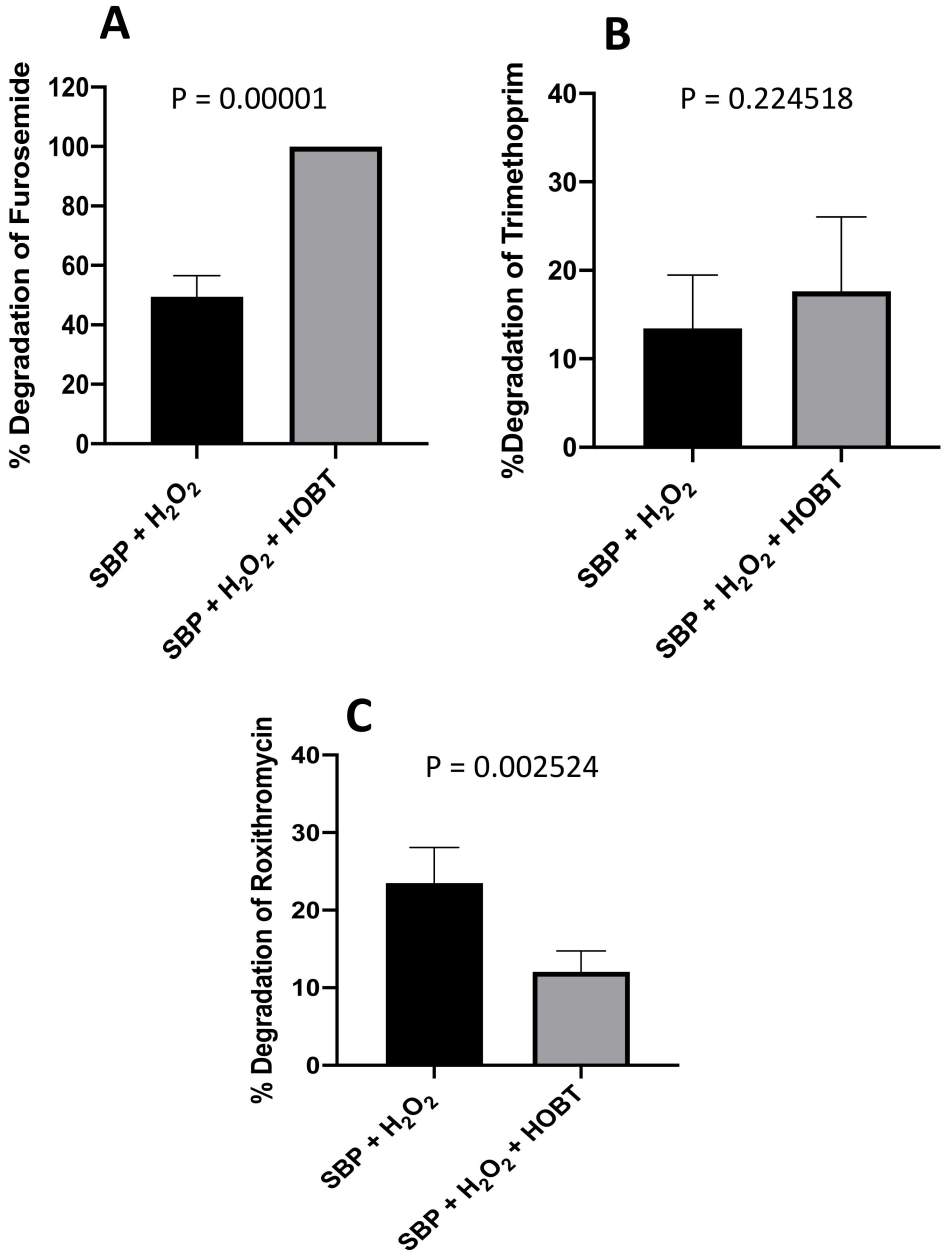
Percentage degradation of (**A**) furosemide; (**B**) trimethoprim; and (**C**) roxithromycin in the presence and absence of the redox mediator (HOBT). [Pollutants] = 2 ppm, [Enzyme] = 0.39 μM, [H_2_O_2_] = 0.3 mM (0.1 mM added 3 times of 10 min interval), [HOBT] = 0.1 mM, pH = 4.

Redox mediators (RMs) are small organic molecules that act as facilitators in oxidoreductase-mediated reactions [20,35]. We and others have previously shown that the presence of a redox mediator in the peroxidase-based reaction may affect the organic pollutant degradation in different ways. These RMs may increase the degradation of the pollutants, inhibit their degradation, or have no effect at all. Therefore, the role of 1-hydroxybenzotriazole (HOBT), a commonly used redox mediator was investigated on the efficiency of SBP-assisted degradation of the chosen 21 Eps. Although HOBT is known to have aquatic toxicity [36], it is used here only as a model redox mediator, and will need to be replaced with a non-toxic compound when used for real-life wastewater treatment applications. Figure 3 summarizes the three different effects of HOBT on the degradation of 21 emerging pollutants tested in this study. For example, it seems from Figure 3A that the presence of a redox mediator significantly enhanced the degradation of the furosemide pollutant. SBP alone was able to degrade about 50% of furosemide, whereas the percentage of degradation reached 100% by the addition of HOBT in the reaction mixture. Similar findings have also been documented earlier [19], where the addition of HOBT led to a dramatic increase in the degradation of various emerging pollutants by HRP, LiP, chloroperoxidase, and SBP. On the other hand, the inclusion of HOBT had almost no effect on the reaction of trimethoprim as the percentage of degradation of trimethoprim almost remained similar (~15%) with and without HOBT (Figure 3B).

The results with roxithromycin showed the inhibitory effect of HOBT on the degradation efficiency of the SBP-catalyzed reaction (Figure 3C). In the absence of HOBT, 23.5% of roxithromycin was degraded; however, the degradation decreased to 12% by the addition of HOBT. We have recently published similar results showing complete inhibition of MnP-mediated degradation of thiabendazole in the presence of HOBT [19]. Similarly, chloroperoxidase-catalyzed chlorination of thiabendazole was also dramatically decreased in the presence of HOBT [33]. We speculate that the inhibitory effect of HOBT for some emerging pollutants is most likely due to the competition of HOBT for the emerging pollutant-binding site on the peroxidases.

The degradation of various emerging pollutants by SBP in the presence and absence of HOBT is summarized in Table 1. A closer analysis of the degradation data allows them to be categorized into five distinct groups; Group A shows pollutants that were degraded equally efficiently in the presence or absence of HOBT; Group B shows enhanced degradation of pollutants when HOBT was added to the reaction; Group C shows lower degradation efficiencies of pollutants in the presence of HOBT; Group D pollutants shows similar (low) degradation of pollutants with and without HOBT; while Group E (11 pollutants) shows no significant degradation (percentages of degradation less than 10) under any condition. 

### 3.3. Characterization of Immobilized Hybrid Biocatalysts

SBP was immobilized onto TiO_2_ and ZnO by following a previously reported procedure [30]. As can be seen from the SEM photographs, TiO_2_ and ZnO had different morphologies, with ZnO being substantially bigger than TiO_2_ nanoparticles. Interestingly and in contrast to TiO_2_, immobilization of SBP on ZnO, caused a big dramatic change in the structural morphology of this photocatalyst (Figure S1). The crystalline structure of all the samples (photocatalysts, APTES-functionalized photocatalysts, and SBP-hybrid biocatalysts) was examined using a Shimadzu-6100 X-ray powder diffractometer with Cu-Kα radiation, with the data collected from 10°–70° at a rate of 2°/min (Supplementary Figure S2). Functionalization of TiO_2_ with APTES and subsequent glutaraldehyde-mediated immobilization of SBP onto it did not cause any changes in the XRD pattern, as compared to the standard XRD spectrum that is usually observed with neat TiO_2_ (Wei et al., 2013). However, as seen with the XRD analysis, immobilization of SBP onto ZnO using glutaraldehyde indicated a strong interaction/reaction between ZnO and SBP (Figure S2). Taken together, both SEM and XRD are consistent with a strong interaction between ZnO and glutaraldehyde-mediated immobilized SBP, showing different shapes and crystallinities for ZnO-SBP as compared with neat ZnO. The very different XRD pattern observed for ZnO-SBP closely resembles the XRD spectra of “Zinc acetate dihydrate” [37]. However, the reason for this reaction and change in XRD pattern is unknown and requires further studies. The successful immobilization of SBP onto the photocatalysts was confirmed by comparing the FTIR spectra of TiO_2_-SBP and ZnO-SBP hybrid catalysts with neat TiO_2_ or ZnO (Supplementary Figure S3). As can be seen in panels A-C (Figure S3), immobilization of SBP on TiO_2_ led to the appearance of a new peak around 1200 cm^−1^, corresponding to the C-N vibration, typically observed when proteins are immobilized on TiO_2_ [38]. The same C-N peak was also observed for ZnO-SBP sample (panel F, Figure S3), in addition to the “Amide I” C = O peak (from SBP) at around 1650 cm^−1^, as well as the N-H (stretch) around 3400 cm^−1^ [39,40], thus confirming the immobilization of SBP onto ZnO as well. The other expected characteristics peaks for the APTES-mediated functionalization of ZnO and TiO_2_ (e.g., the N-H (bending) peaks around 1550 cm^−1^) are also indicated (panel E).

### 3.4. Degradation of 21 Eps by the Immobilized Hybrid Biocatalysts

The degradation of these 21 Eps was also tested by using immobilized SBP on TiO_2_ and ZnO with and without the addition of the redox mediator, HOBT. Table 2 summarizes the results obtained by the TiO_2_-SBP treatment of 21 Eps. It was found that TiO_2_-SBP + H_2_O_2_ alone (without redox mediator) resulted in the complete and rapid degradation of caffeic acid and MBT (within 30 min). It also showed efficient degradation (60.2%) of caffeine. However, furosemide, roxithromycin, cimetidine, meloxicam, ibuprofen, and norfloxacin were only partially degraded with percentages ranging from 15% to 45%. The addition of HOBT enhanced the TiO_2_-SBP + H_2_O_2_ of some pollutants. For example, it resulted in complete degradation of furosemide, (from 15.7% in the absence of HOBT). Similarly, it enhanced the degradation of meloxicam from only 26.8% (in the absence of HOBT) to 99.8% after the addition of HOBT. The degradation of other pollutants that are listed in Group B was also improved by the addition of the redox mediator. On the contrary, the degradation of norfloxacin was decreased following the addition of HOBT to the reaction mixture, indicating an inhibitory effect of a redox mediator. Other pollutants had similar degradation efficiencies by TiO_2_-SBP in the presence or absence of HOBT, such as ibuprofen, cimetidine, and roxithromycin. The TiO_2_-SBP hybrid biocatalyst failed to degrade 7 pollutants out of the 21 pollutants tested, even in the presence of HOBT (Group E). 

The degradation of these selected 21 emerging pollutants was also studied using the ZnO-SBP hybrid-catalyst. Table 3 summarizes the results from the ZnO-SBP-mediated degradation of our panel of emerging pollutants. As can be seen from the table, ZnO-SBP + H_2_O_2_ alone was able to completely degrade MBT, as well as significant amounts of caffeic acid (54.4%) and roxithromycin (48.6%). As seen previously, the addition of the redox mediator, HOBT, led to a significant enhancement of enzyme-mediated degradation, resulting in complete degradation of caffeic acid and meloxicam. Although ZnO-SBP without HOBT was not able to degrade furosemide significantly, the addition of HOBT led to its dramatic and complete degradation. Other pollutants that are listed in Group B also showed higher degradation by the addition of HOBT. However, the degradation of MCPA was inhibited due to the inclusion of the HOBT. Pollutants listed in Group D appeared to be degraded similarly in the presence and absence of the redox mediator. Out of the 21 tested emerging pollutants, 6 pollutants (listed in group E) appeared to be recalcitrant as ZnO-SBP alone or in the presence of HOBT could not degrade any of them (Table 3).

### 3.5. Comparison between Free vs. Immobilized Enzyme

The ability of immobilized enzymes was compared to the respective free counterparts in terms of degrading the 21 chosen emerging pollutants. Table 4 summarizes all the data obtained when a mixture of 21 Eps was treated with free SBP as well as immobilized SBP composites (TiO_2_-SBP and ZnO-SBP). For the sake of simplicity, we focused only on the Eps that were degraded by more than 10% with either the free or immobilized SBP hybrid biocatalysts. The free SBP enzyme degraded 10 pollutants out of the 21 tested pollutants, while TiO_2_-SBP degraded 14 pollutants and ZnO-SBP degraded 15 pollutants. Therefore, it appears that immobilized enzyme-photocatalyst hybrids showed a better capability of degrading pollutants (in terms of the number of degraded pollutants). Pollutants including roxithromycin, caffeine, ibuprofen, hydrochlorothiazide, DEET, venlafaxine-HCl, MCPA, phenytoin, lincomycin-HCl, norfloxacin, and cimetidine had better degradation rates when treated by the immobilized SBP (either TiO_2_-SBP or ZnO-SBP), as compared to free SBP. It is noteworthy that some of these pollutants were recalcitrant to degradation by the free enzyme and were only degraded by the immobilized SBP-catalyzed reaction, e.g., free SBP could not degrade norfloxacin but ZnO-SBP resulted in 45.1% degradation. Furthermore, a significant difference was recorded between the immobilized and un-immobilized SBP for the degradation of some Eps such as caffeine and norfloxacin. Free SBP degraded 21.8% of caffeine, while TiO_2_-SBP led to 60.2% degradation of caffeine, which was three-times greater than the free enzyme. The better degradation of some Eps by immobilized SBPs could be due to the synergistic effect of the radicals generated by SBP enzyme and the photocatalysts, as has been previously reported by Calza et al. [41,42], when they used a similar SBP-TiO_2_ hybrid system, or Sarro et al. when they immobilized SBP onto ZnO-based materials [43].

Surprisingly, SMX showed a better degradation by free SBP compared to the immobilized hybrid biocatalysts, where 99.3% of SMX was degraded by free SBP while ZnO-SBP and TiO_2_-SBP catalyzed 32% and 38% degradation of SMX, respectively. This could be due to the possibility that sometimes glutaraldehyde-based immobilization can lead to partially denatured or conformationally constrained enzymes, resulting in the loss of enzyme activity [44]; however, this hypothesis needs to be further tested. A number of Eps showed equally similar degradation either by free SBP or immobilized form, such as MBT, meloxicam, caffeic acid, and furosemide (Table 4). Out of the 21 tested Eps, three of the pollutants, namely prometryn, fluometuron, and thiabendazole were recalcitrant to degradation by both free enzyme and immobilized enzyme. As mentioned in Section 2.2, although about twice as much SBP was immobilized onto TiO_2_ support (per unit mass) as compared to ZnO, this did not appear to have a significant effect on the degradation abilities of the two hybrid biocatalysts. This is not surprising, as the anatase form of TiO_2_ and ZnO has similar band gaps of 3.2 eV and 3.4 eV, respectively, and hence they are both capable of degrading organic pollutants, albeit ZnO appears to be slightly better due to better absorption of light/UV [45]. For example, MBT seemed to be degraded equally well by both TiO_2_-SBP and ZnO-SBP, as were meloxicam, caffeic acid, and furosemide. Furthermore, it seemed that roxithromycin was better degraded by ZnO-SBP (which had less SBP immobilized on it) than TiO_2_-SBP.

**Figure 4:**
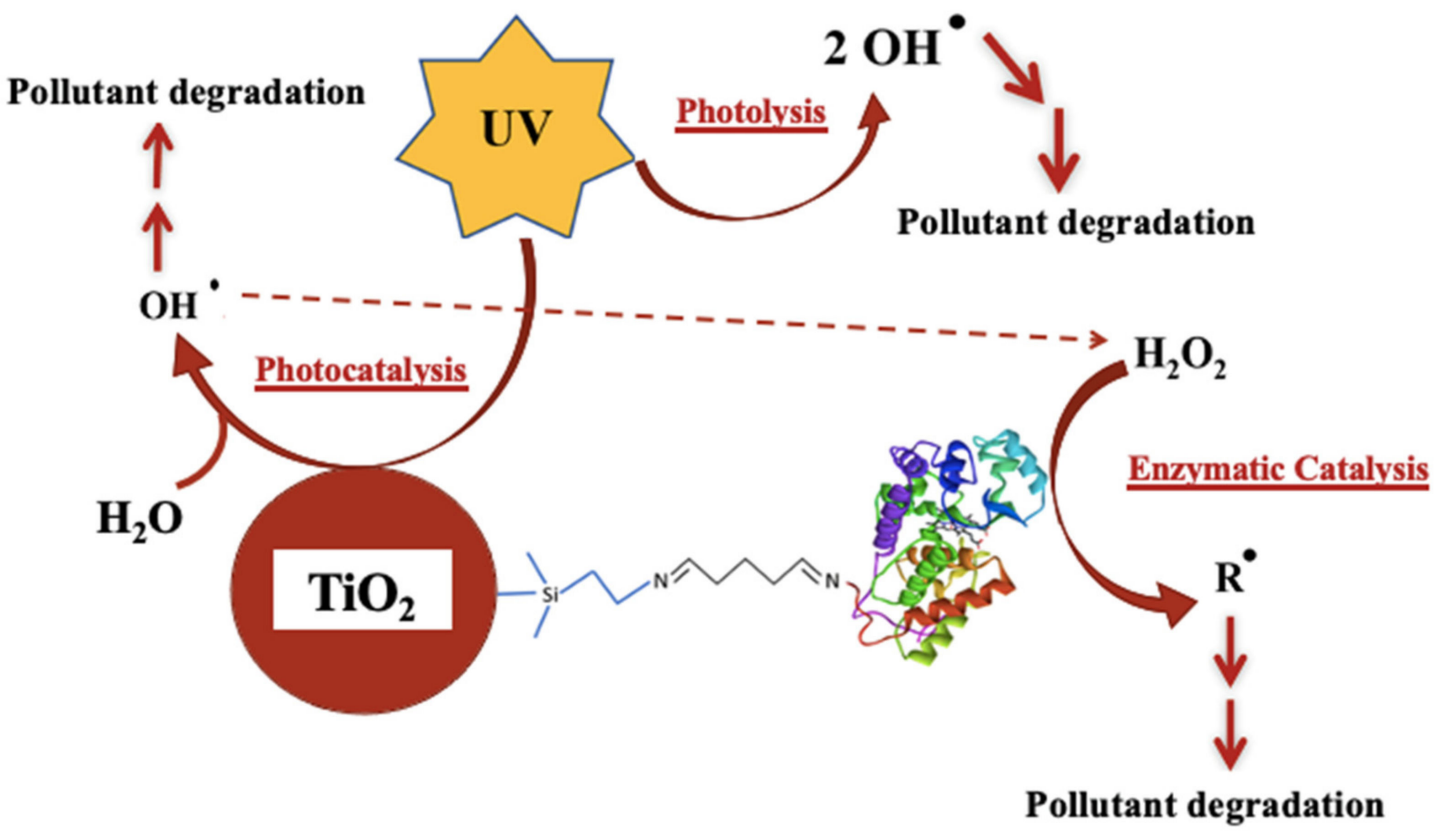
Schematic representation of reactions involved when a hybrid catalyst is used.

**Figure 5:**
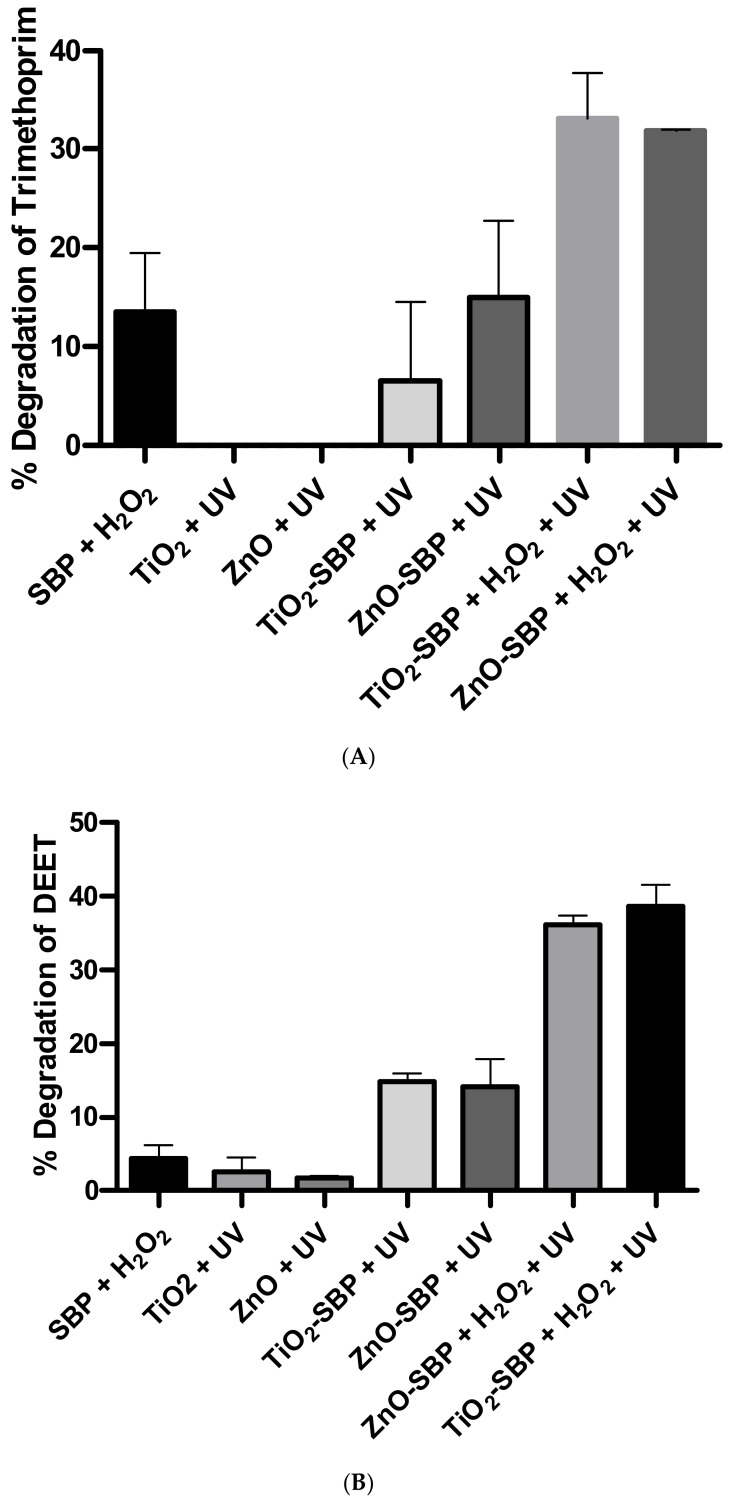
Percentage degradation of (**A**) Trimethoprim; and (**B**) DEET. Experimental conditions were as per Materials and Methods.

Since the solid supports used for the immobilization of SBP were photocatalytic, we wanted to see if we could combine the remediation powers of the TiO_2_/ZnO photocatalyst and our peroxidase (SBP) to potentially create a doubly powerful hybrid remediation catalyst, as schematically shown in Figure 4. As suggested, in the scheme, exposure to UV would lead the production of reactive hydroxyl radicals by the photocatalysts (TiO_2_ or ZnO), which could combine to produce H_2_O_2_, which in turn could be used by SBP or split by UV light to produce additional radicals. The resulting “triple sources” of hydroxyl or other radicals could, in theory, lead to faster degradation of some organic pollutants. Our preliminary results suggest that for at least two of the emerging pollutants we tested, this may be in fact the case. As can be seen in Figure 5 both photocatalysts (TiO_2_ or ZnO), when used alone (neat), did not cause any significant degradation of trimethoprim (or DEET). However, they were degraded slightly (<15%) by incubating with TiO_2_-SBP and ZnO-SBP and UV light. Interestingly, treating these two recalcitrant pollutants with TiO_2_-SBP and ZnO-SBP in the presence of H_2_O_2_ together with exposure to UV light resulted in a dramatic and significant enhancement in degradation (more than 30%). These results suggest that immobilization of SBP onto photocatalytic supports could give better degradation of recalcitrant pollutants compared to using free enzymes or neat photocatalysts alone. This can be due to the combined synergistic effect of the H_2_O_2_-peroxidase system, UV-H_2_O_2_ photolytic oxidation as well as UV-photocatalytic-mediated oxidation of trimethoprim and DEET. Again, these preliminary results are highly suggestive that photocatalyst-peroxidase hybrid catalysts could be unusually powerful remediation agents. The results shown here are consistent with a previously published study on TiO_2_-SBP composite catalysts which showed synergistic results of SBP and TiO_2_ for the degradation of 2,4,6-trichlorophenol [42]. Another recent study by [43] has shown similar enhanced degradation by SBP when immobilized on nanofibers containing doped TiO_2_ and ZnO photocatalysts. This study considered a panel of six different emerging pollutants (namely diclofenac, naproxen, iopamidol, imidacloprid, bisphenol A, and 2,4-dichlorophenol). Our current study is a further confirmation of this approach of combining enzymatic and photocatalytic approaches but has also expanded this principle to a much larger panel of 21 different emerging pollutants.

### 3.6. Reusability of Immobilized Enzyme

**Figure 6:**
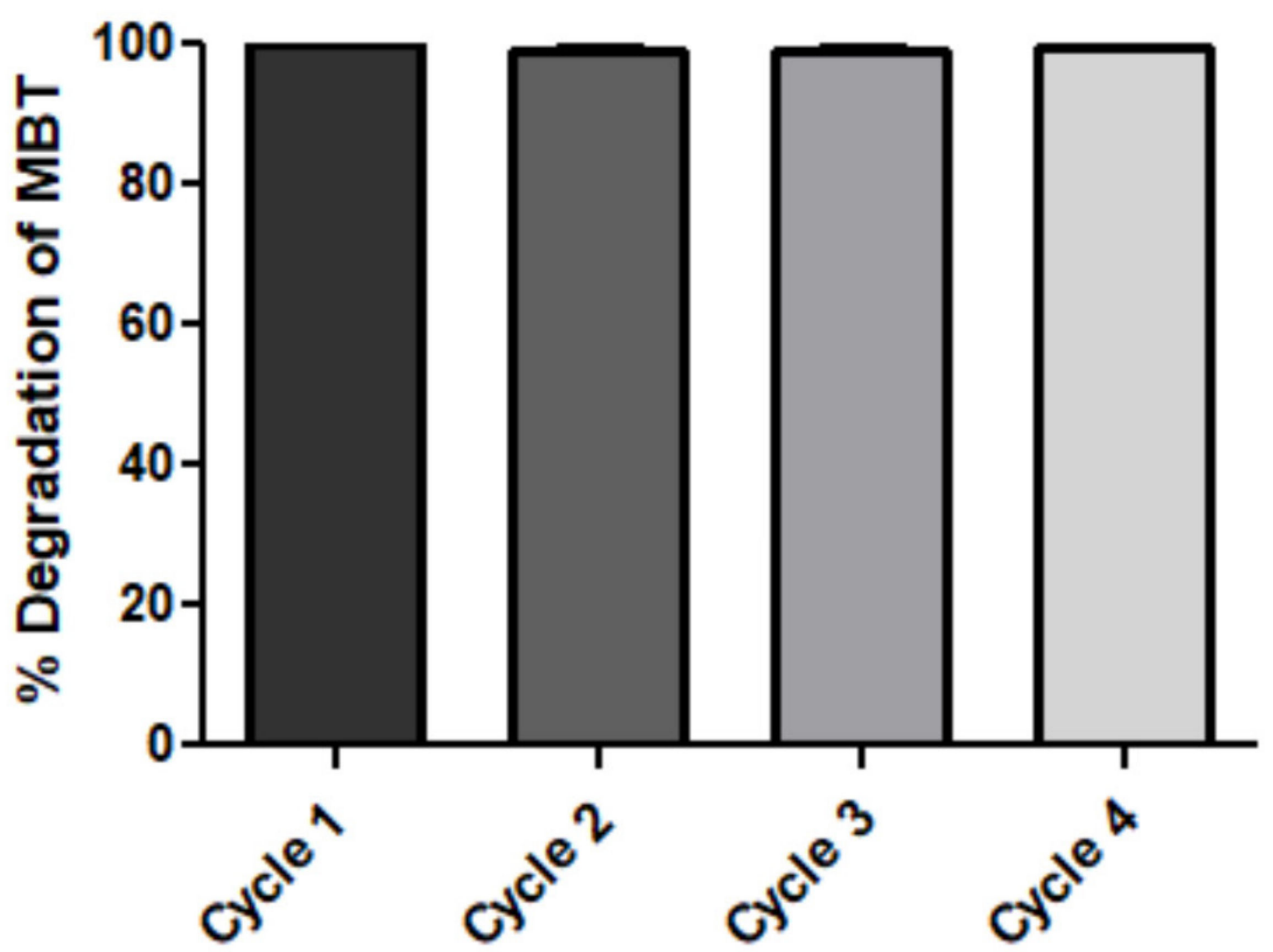
Recyclability of TiO_2_-SBP. After four consecutive degradation cycles of MBT by TiO_2_-SBP, the degradation efficiency of MBT was up to 95%. [MBT]= 2 ppm, 20 mg of TiO_2_-SBP, [H_2_O_2_]= 0.6 mM (0.2 mM added 3 times of 10 min interval), [HOBT]= 0.1 mM, pH=4.

One of the rationales for immobilizing SBP onto solid supports was to allow for the recycling of the enzyme and hence the potential real-life application. Figure 6 shows the recycling ability of TiO_2_-SBP hybrid biocatalyst in degrading MBT, tested in four consecutive cycles. As can be seen, in each cycle, MBT was efficiently degraded by TiO_2_-SBP + H_2_O_2_ + HOBT, with the TiO_2_-SBP hybrid biocatalyst retaining up to 95% degradation efficiency of MBT. A slight reduction in degradation activity of the enzymes is generally observed with the recycling of immobilized enzymes, presumably due to leaching of the enzyme or potential denaturation [46]. In addition, the solution microenvironment might also cause alterations in the enzyme’s conformational integrity during the recycling process, leading to a slight loss of the enzyme activity [47]. However, our results suggest that the functionalized TiO_2_ photocatalytic support allowed for the recycling of SBP for at least four consecutive degradation cycles without any significant loss of enzyme activity. Thus suggesting that glutaraldehyde-immobilized SBP onto TiO_2_ prevented any leaching of the enzyme and that SBP maintained its activity after covalently linked to the TiO_2_ support.

## 4. Concluding Remarks

In summary, SBP could be immobilized via covalent linkages onto two different photocatalytic supports (TiO_2_ and ZnO) to develop new hybrid biocatalysts that showed similar thermal stability as the free enzyme (Figure S4). However, there was a slight shift in the pH optimum for SBP upon immobilization (Figure S4), which is often seen when enzymes are immobilized onto solid supports [48]. Interestingly, TiO_2_- and ZnO-immobilized SBP presented better degradation efficiency than the free SBP, and many of the emerging pollutants including roxithromycin, meloxicam, norfloxacin, cimetidine, ibuprofen, caffeine, MBT, and hydrochlorothiazide were better degraded by TiO_2_-SBP or ZnO-SBP as compared with the free SBP. The results presented here showed that SBP immobilization onto photocatalytic supports not only allowed for efficient recycling of the enzyme, but also created a potential hybrid catalyst, much more powerful than either the free enzyme or the free photocatalysts. In conclusion, the high pollutant degradation ability of the newly developed hybrid biocatalysts shows strong potential for environmental remediation and biotechnology.

## Figures and Tables

**Table 1:** Degradation of 21 Eps by SBP ± HOBT.

		Emerging Pollutant	SBP	SBP + HOBT
			% Degradation	% Degradation
Group A	1	MBT	**99.4 ± 0.1**	**98.7 ± 0.3**
	2	Meloxicam	**98.8 ± 0.2**	**99.0 ± 0.3**
	3	Caffeic acid	**88.2 ± 2.4**	**100.0 ± 0.1**
Group B	4	Furosemide	49.5 ± 7.0	**100.0 ± 0.1**
	5	SMX	38.5 ± 6.9	**99.3 ± 0.3**
Group C	6	Roxithromycin	23.5 ± 4.6	12.0 ± 2.7
Group D	7	Caffeine	16.7 ± 8.5	21.8 ± 1.3
	8	Ibuprofen	16.6 ± 5.3	13.8 ± 8.9
	9	Atenolol	13.5 ± 5.8	6.9 ± 9.5
	10	Trimethoprim	13.4 ± 6.0	17.6 ± 8.4
Group E	11	Prometryn	8.1 ± 7.3	4.3 ± 5.6
	12	Hydrochlorothiazide	5.9 ± 3.4	5.5 ± 5.3
	13	DEET	4.4 ± 1.7	5.4 ± 1.4
	14	Venlafaxine-HCl	4.4 ± 3.1	3.8 ± 2.4
	15	MCPA	4.1 ± 6.4	6.1 ± 6.3
	16	Phenytoin	3.7 ± 6.7	0.0 ± 4.2
	17	Lincomycin-HCl	2.6 ± 7.9	5.8 ± 8.1
	18	Norfloxacin	2.5 ± 4.8	5.5 ± 3.6
	19	Cimetidine	2.1 ± 7.8	8.7 ± 7.7
	20	Fluometuron	1.8 ± 4.6	2.5 ± 4.5
	21	Thiabendazole	0.0 ± 0.1	4.3 ± 3.9

**Table 2:** Degradation of 21 Eps by TiO_2_-SBP ± HOBT.

		Emerging Pollutant	TiO_2_-SBP	TiO_2_-SBP + HOBT
			% Degradation	% Degradation
Group A	1	Caffeic acid	**100.0 ± 0.1**	**100.0 ± 0.1**
	2	MBT	**100.0 ± 0.1**	**100.0 ± 0.1**
	3	Caffeine	60.2 ± 4.1	57.2 ± 4.6
Group B	4	Meloxicam	26.8 ± 4.4	**99.8 ± 0.1**
	5	Furosemide	15.7 ± 5.5	**100.0 ± 0.1**
	6	SMX	9.7 ± 1.5	37.9 ± 3.2
	7	DEET	3.7 ± 2.8	17.6 ± 2.8
	8	MCPA	3.1 ± 3.8	11.5 ± 7.1
	9	Lincomycin-HCl	2.8 ± 1.4	15.1 ± 4.9
	10	Hydrochlorothiazide	0.0 ± 0.1	14.3 ± 7.5
Group C	11	Norfloxacin	45.1 ± 1.3	0.0 ± 0.1
Group D	12	Ibuprofen	32.2 ± 8.0	39.5 ± 5.3
	13	Cimetidine	24.4 ± 2.0	19.3 ± 2.5
	14	Roxithromycin	24.4 ± 7.3	17.7 ± 3.4
Group E	15	Prometryn	9.4 ± 5	9.2 ± 5.4
	16	Venlafaxine-HCl	6.2 ± 2.5	6.5 ± 5.5
	17	Fluometuron	5.7 ± 8.9	3.0 ± 9.6
	18	Trimethoprim	3.9 ± 3.8	7.0 ± 3.5
	19	Phenytoin	3.2 ± 1.9	7.4 ± 2.5
	20	Thiabendazole	0.2 ± 2.8	4.5 ± 3.2
	21	Atenolol	0.0 ± 0.1	0.2 ± 4.2

**Table 3:** Degradation of 21 Eps by ZnO-SBP ± HOBT.

		Emerging Pollutant	ZnO-SBP	ZnO-SBP + HOBT
			% Degradation	% Degradation
**Group A**	**1**	MBT	**100.0 ± 0.1**	**100.0 ± 0.1**
Group B	2	Caffeic acid	54.4 ± 3.8	**100.0 ± 0.1**
	3	Hydrochlorothiazide	19.0 ± 1.5	25.8 ± 3.7
	4	SMX	12.5 ± 2.4	31.9 ± 4.1
	5	Meloxicam	11.4 ± 6.9	**100.0 ± 0.1**
	6	Lincomycin-HCl	5.6 ± 3.6	14.9 ± 3.5
	7	Cimetidine	4.7 ± 1.0	11.3 ± 4.4
	8	DEET	3.0 ± 5.5	14.4 ± 2.2
	9	Furosemide	2.6 ± 7.1	**100.0 ± 0.1**
Group C	10	MCPA	18.7 ± 7.8	1.6 ± 9.8
Group D	11	Roxithromycin	48.6 ± 4.9	37.4 ± 8.6
	12	Trimethoprim	13.8 ± 9.4	17.8 ± 1.2
	13	Phenytoin	13.4 ± 3.3	10.3 ± 2.8
	14	Ibuprofen	13.1 ± 6.4	22.2 ± 8.8
	15	Venlafaxine-HCl	7.3 ± 5.7	11.0 ± 4.5
Group E	16	Prometryn	5.6 ± 1.9	4.0 + 3.9
	17	Norfloxacin	5.5 ± 3.0	5.5 ± 1.7
	18	Thiabendazole	4.8 ± 1.1	6.5 ± 1.4
	19	Fluometuron	2.8 ± 6.8	2.2 ± 7.1
	20	Caffeine	0.2 ± 9.6	1.5 ± 7.4
	21	Atenolol	0.0 ± 0.1	9.5 ± 4

**Table 4:** Summary of % degradation data when a mixture of 21 Eps was treated with free SBP and immobilized SBP (TiO_2_-SBP and ZnO-SBP). The values in bold show the highest degradation obtained by either the free enzyme or immobilized enzymes.

	Emerging Pollutant	SBP		SBP-TiO_2_		SBP-ZnO	
		No HOBT	With HOBT	No HOBT	With HOBT	No HOBT	With HOBT
1	MBT	99.4 ± 0.1	98.7 ± 0.3	**100.0** ± 0.1	**100.0** ± 0.1	**100.0** ± 0.1	**100.0** ± 0.1
2	Meloxicam	98.8 ± 0.2	99.0 ± 0.3	26.8 ± 4.4	**99.8** ± 0.1	11.4 ± 6.9	**100.0** ± 0.1
3	Caffeic acid	88.2 ± 2.4	**100.0** ± 0.1	**100.0** ± 0.1	**100.0** ± 0.1	54.4 ± 3.8	**100.0** ± 0.1
4	Furosemide	49.5 ± 7.0	**100.0** ± 0.1	15.7 ± 5.5	**100.0** ± 0.1	2.6 ± 7.1	**100.0** ± 1.0
5	SMX	38.5 ± 6.9	**99.3** ± 0.3	9.7 ± 1.5	37.9 ± 3.2	12.5 ± 2.4	31.9 ± 4.1
6	Roxithromycin	23.5 ± 4.6	12.0 ± 2.7	24.4 ± 7.3	17.7 ± 3.4	**48.6** ± 4.9	37.4 ± 8.6
7	Caffeine	16.7 ± 8.5	21.8 ± 1.3	**60.2** ± 4.1	57.2 ± 4.6	0.2 ± 9.6	1.5 ± 7.4
8	Ibuprofen	16.6 ± 5.3	13.8 ± 8.9	32.2 ± 8.0	**39.5** ± 5.3	13.1 ± 6.4	22.2 ± 8.8
9	Atenolol	**13.5** ± 5.8	6.9 ± 9.5	0.0 ± 0.1	0.2 ± 4.2	0.0 ± 0.1	9.5 ± 4.0
10	Trimethoprim	13.4 ± 6.0	**17.6** ± 8.4	3.9 ± 3.8	7.0 ± 3.5	13.8 ± 9.4	**17.8** ± 1.2
11	Prometryn	8.1 ± 7.3	4.3 ± 5.6	9.4 ± 5.0	9.2 ± 5.4	5.6 ± 1.9	4.0 ± 3.9
12	Hydrochlorothiazide	5.9 ± 3.4	5.5 ± 5.3	0.0 ± 0.1	14.3 ± 7.5	19.0 ± 1.5	**25.8** ± 3.7
13	DEET	4.4 ± 1.7	5.4 ± 1.4	3.7 ± 2.8	**17.6** ± 2.8	3.0 ± 5.5	14.4 ± 2.2
14	Venlafaxine-HCl	4.4 ± 3.1	3.8 ± 2.4	6.2 ± 2.5	6.5 ± 5.5	7.3 ± 5.7	**11.0** ± 4.5
15	MCPA	4.1 ± 6.4	6.1 ± 6.3	3.1 ± 3.8	11.5 ± 7.1	**18.7** ± 7.8	1.6 ± 9.8
16	Phenytoin	3.7 ± 6.7	0.0 ± 4.2	3.2 ± 1.9	7.4 ± 2.5	**13.4** ± 3.3	10.3 ± 2.8
17	Lincomycin-HCl	2.6 ± 7.9	5.8 ± 8.1	2.8 ± 1.4	**15.1** ± 4.9	5.6 ± 3.6	**14.9** ± 3.5
18	Norfloxacin	2.5 ± 4.8	5.5 ± 3.6	**45.1** ± 1.3	0.0 ± 0.1	5.5 ± 3.0	5.5 ± 1.7
19	Cimetidine	2.1 ± 7.8	8.7 ± 7.7	**24.4** ± 2.0	19.3 ± 2.5	4.7 ± 1.0	11.3 ± 4.4
20	Fluometuron	1.8 ± 4.6	2.5 ± 4.5	5.7 ± 8.9	3.0 ± 9.6	2.8 ± 6.8	2.2 ± 7.1
21	Thiabendazole	0.0 ± 0.1	4.3 ± 3.9	0.2 ± 2.8	4.5 ± 3.2	4.8 ± 1.1	6.5 ± 1.4

## Data Availability

Not applicable.

## Supplementary Materials

The following are available online at https://www.mdpi.com/article/10.3390/biom11060904/s1, Figure S1: Scanning electron microscope (SEM) images of pure photocatalysts (TiO_2_ and ZnO), functionalized photocatalysts (TiO_2_-APTES and ZnO-APTES) and immobilized SBP enzyme on the photocatalysts (TiO_2_-SBP and ZnO-SBP). Figure S2: X-ray diffraction (XRD) patterns of (A) pure TiO_2_, functionalized TiO_2_ (TiO_2_-APTES) and SBP enzyme immobilized on TiO_2_ (TiO2-SBP) and (B) pure ZnO, functionalized ZnO (ZnO-APTES) and SBP enzyme immobilized on ZnO (ZnO-SBP). Figure S3: Fourier transform infrared spectroscopy (FTIR) spectra of (A) TiO_2_-SBP; (B) TiO_2_-APTES; (C) TiO_2_; (D) ZnO; (E) ZnO-APTES and (F) ZnO-SBP. Figure S4: Influence of pH and temperature on the activity of free SBP and immobilized SBP on TiO_2_ (TiO_2_-SBP). (A) pH 2.0–8.0 and (B) temperature 30–90 °C. Table S1: Summary of MRM mode for the 21 treated emerging pollutants.
