# Supplementary material for: Immobilized Soybean Peroxidase Hybrid Biocatalysts for Efficient Degradation of Various Emerging Pollutants
Source: Biomolecules. 2021 Jun 17;11(6):904. doi: 10.3390/biom11060904 (PMC8235338; doi:10.3390/biom11060904)
Supplement: Supplementary file 1 [file biomolecules-11-00904-s001.zip › biomolecules-1230040-supplementary.pdf]

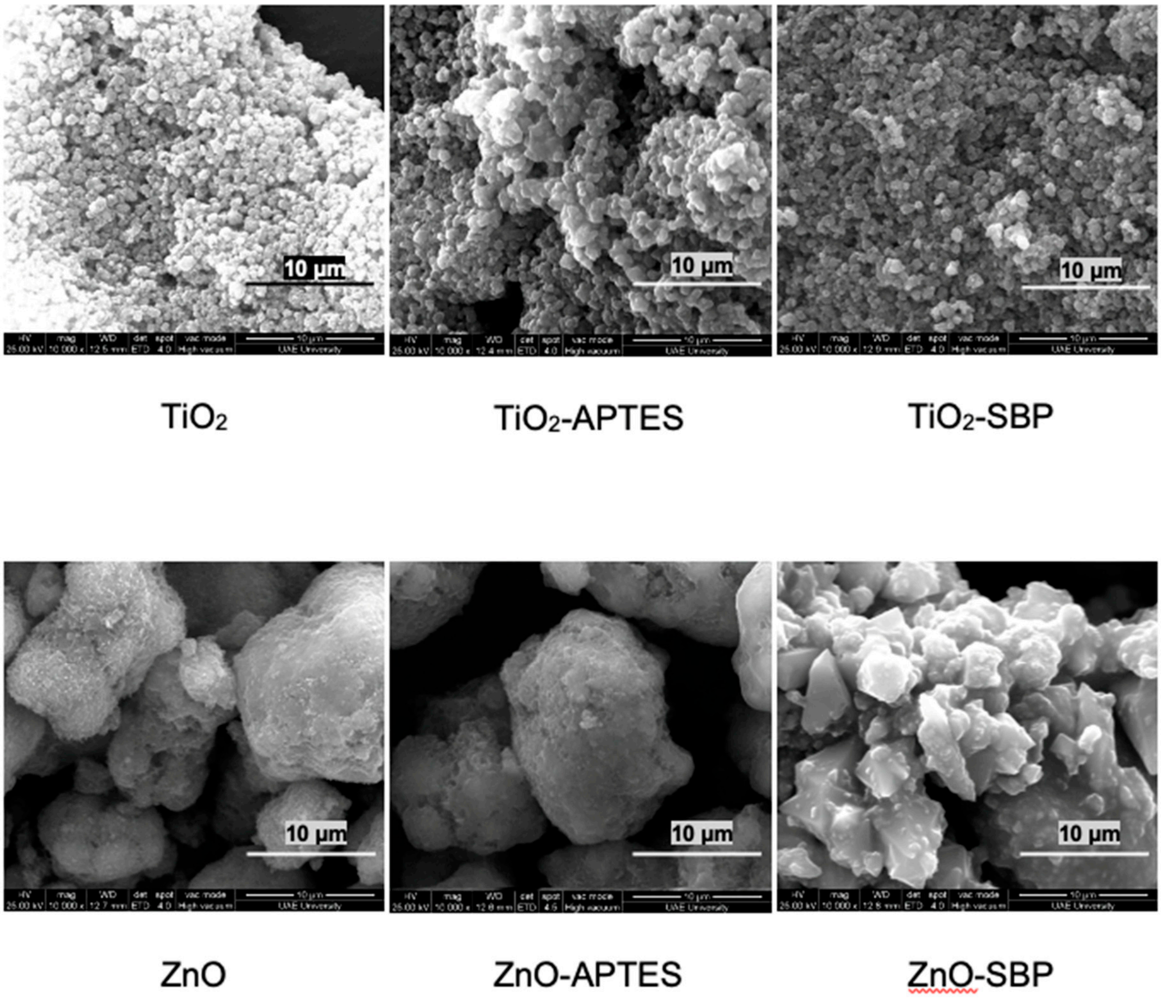

Figure S1: Scanning electron microscope (SEM) images of pure photocatalysts (TiO_2 and ZnO), functionalized photocatalysts (TiO_2-APTES and ZnO-APTES) and immobilized SBP enzyme on the photocatalysts (TiO_2-SBP and ZnO-SBP).

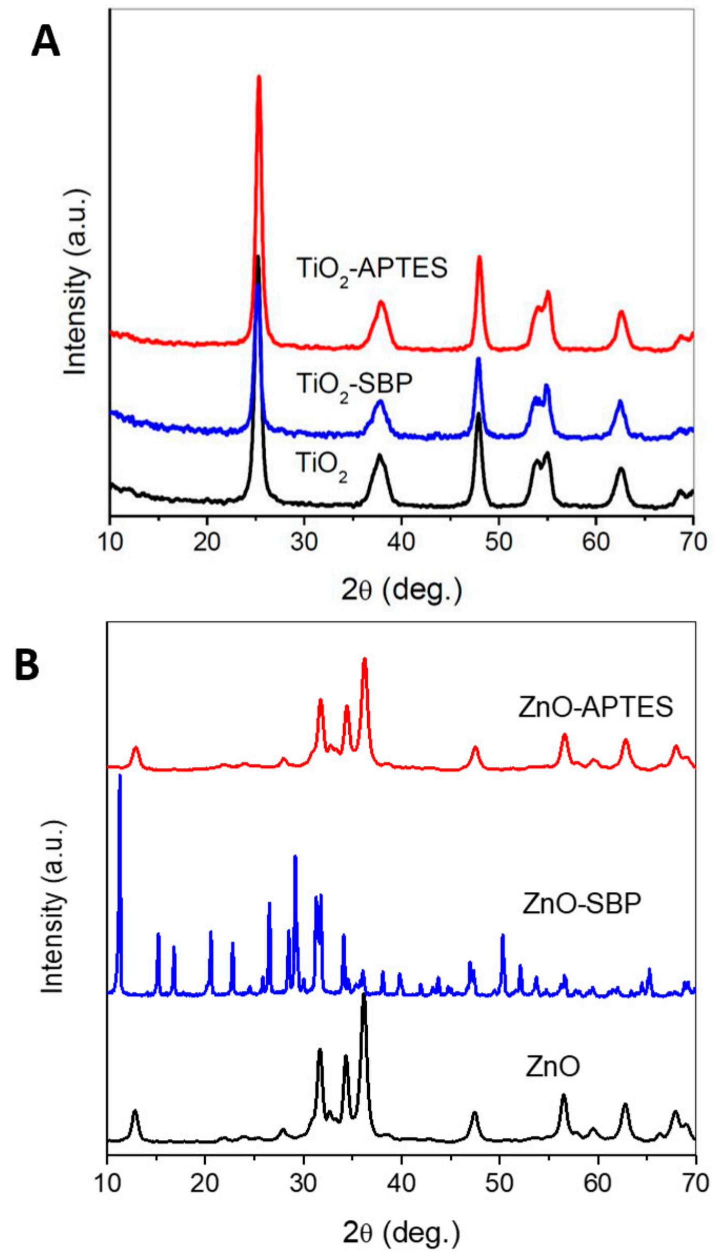

Figure S2: X-ray diffraction (XRD) patterns of (**A**) pure TiO2, functionalized TiO2 (TiO2-APTES) and SBP enzyme immobilized on TiO2 (TiO2-SBP) and (**B**) pure ZnO, functionalized ZnO (ZnO-APTES) and SBP enzyme immobilized on ZnO (ZnO-SBP).

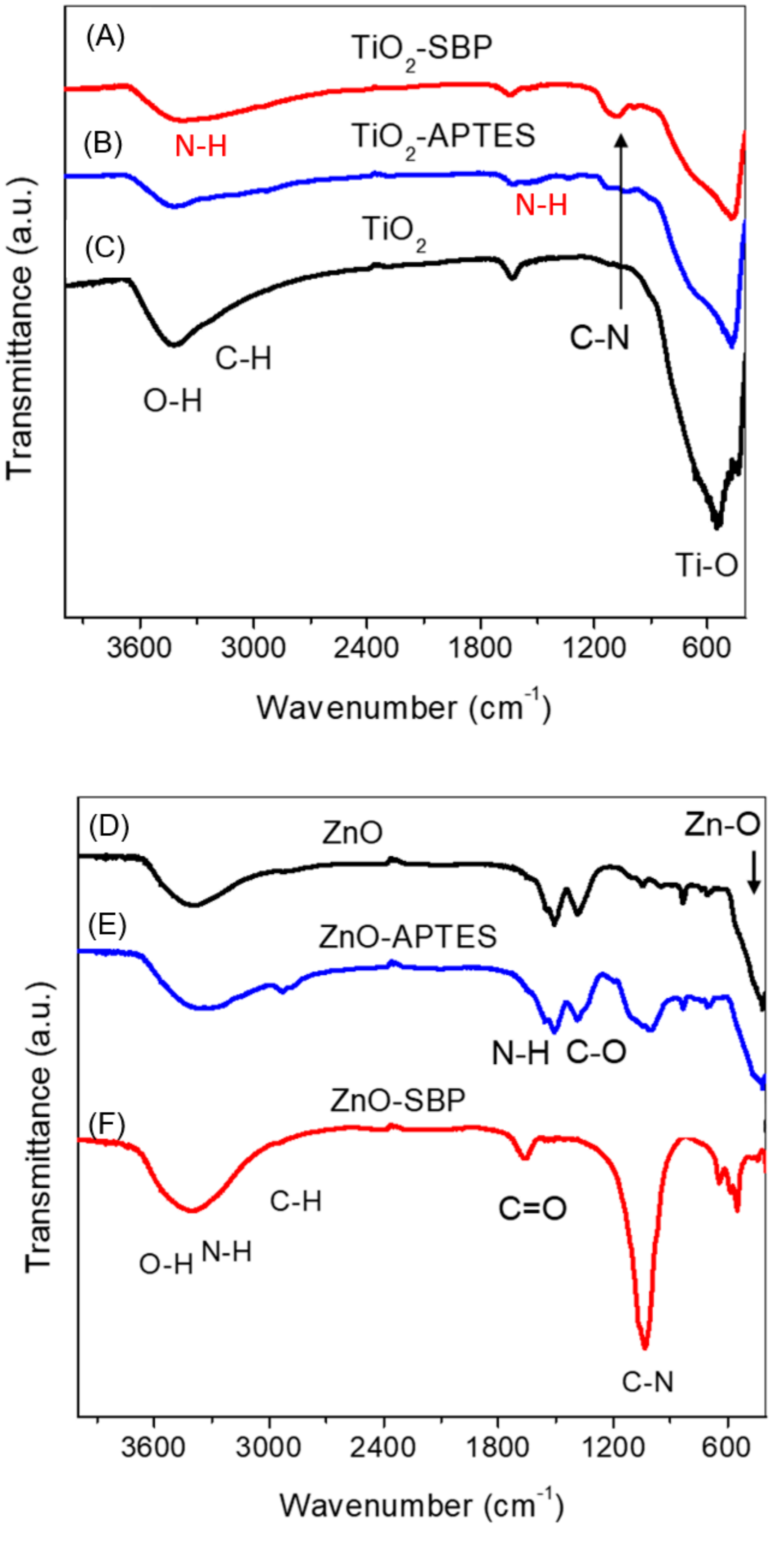

Figure S3: Fourier transform infrared spectroscopy (FTIR) spectra of (**A**) TiO2-SBP; (**B**) TiO2-APTES; (**C**) TiO2; (**D**) ZnO; (**E**) ZnO-APTES and (**F**) ZnO-SBP.

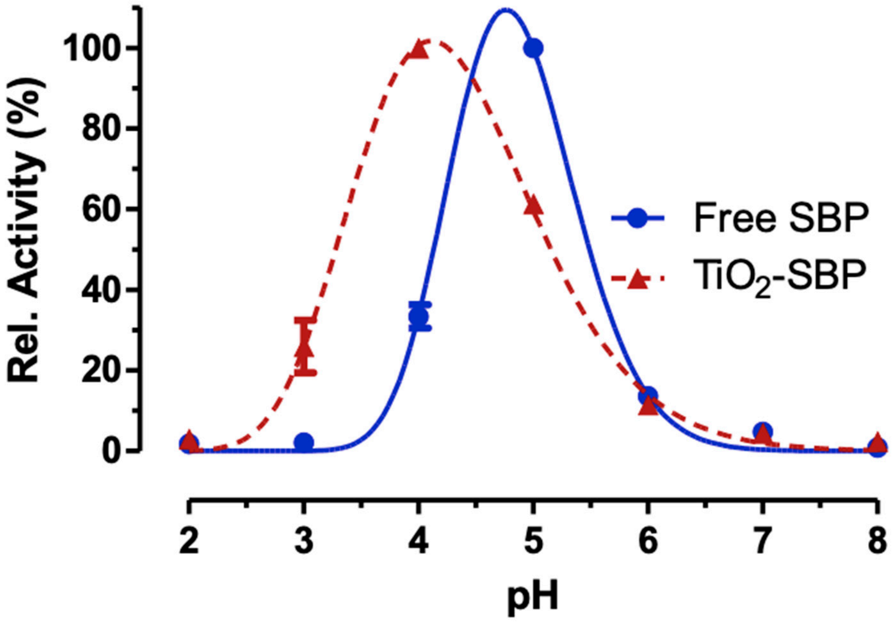

(**A**)

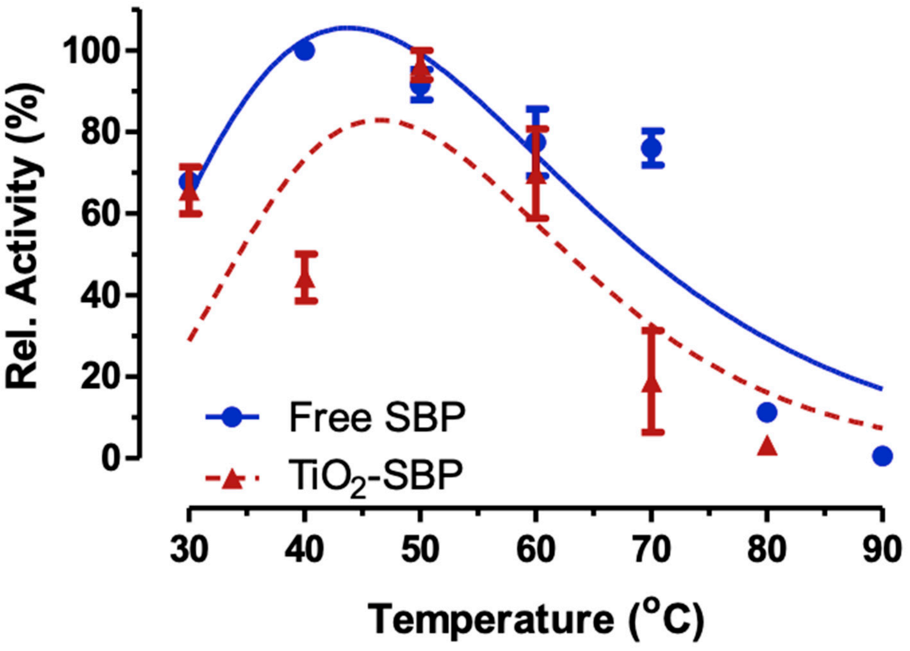

(**B**)

Figure S4: Influence of pH and temperature on the activity of free SBP and immobilized SBP on TiO2 (TiO2-SBP). (**A**) pH 2.0-8.0 and (**B**) temperature 30–90° C.

Table S1. Summary of MRM mode for the 21 treated emerging pollutants.

	Emerging Pollutant	Retention Time (min)	Parent Ion (m/z)	Daughter Ion (m/z)	Polarity	Collision Energy (V)
1	Roxithromycin	11.6	837	680	Positive	20
2	Lincomycin-HCl	7.6	407	359	Positive	20
3	Meloxicam	12.8	352	115	Positive	6
4	Norfloxacin	8.2	320	302	Positive	20
5	Trimethoprim	7.9	291	230	Positive	20
6	Venlafaxine-HCl	9.4	278	260	Positive	10
7	Atenolol	7.1	267	190	Positive	20
8	SMX	9.3	254	156	Positive	20
9	Cimetidine	6.9	253	159	Positive	10
10	Phenytoin	11.1	253	182	Positive	10
11	Prometryn	11.6	242	158	Positive	30
12	Fluometuron	11.7	233	72	Positive	30
13	Ibuprofen	14.4	207	161	Positive	20
14	Thiabendazole	7.6	202	175	Positive	30
15	MCPA	12	201	125	Positive	13
16	Caffeine	7.8	195	138	Positive	30
17	DEET	11.9	192	119	Positive	30
18	Caffeic acid	7.8	181	163	Positive	20
19	MBT	10.6	168	135	Positive	30
20	Furosemide	11	329	285	Negative	15
21	Hydrochlorothiazide	6.4	167	190	Negative	20
